# Small-Satellite System Fault Diagnosis via a Temporal–Spatial 3D-CNN with Imbalanced-Aware Training

**DOI:** 10.3390/s26103116

**Published:** 2026-05-15

**Authors:** Bin Wang, Shu Ting Goh, Sheral Crescent Tissera, Abhishek Rai, Lijie Zhang

**Affiliations:** 1 School of Mechanical Engineering, Yanshan University, Qinhuangdao 066004, China; 2 Department of Electrical and Computer Engineering, National University of Singapore, Singapore 117583, Singapore

**Keywords:** small satellite, fault diagnosis, convolutional neural networks, deep learning

## Abstract

Reliable onboard fault detection and diagnosis (FDD) is essential for autonomous small-satellite constellation operations. The satellite telemetry streams are typically high-dimensional, strongly time-correlated, and severely imbalanced. These characteristics make rare but critical faults hard to recognize. To address these issues, this paper proposes an imbalance-aware spatiotemporal diagnostic framework based on three-dimensional convolutional neural networks (3D-CNNs). Multivariate telemetry is first converted into structured spatiotemporal volumes via sliding-window segmentation and grid-based embedding. This enables the model to jointly learn temporal evolution and cross-parameter coupling patterns. A lightweight residual 3D-CNN is developed to enable end-to-end multi-class classification. In addition, a class-balanced focal objective function is introduced to mitigate class-imbalance issues and enhance sensitivity to minority fault modes. The Lumelite series satellite telemetry dataset, comprising 23 fault types, is constructed for training and evaluation. The proposed lightweight residual 3D-CNN is benchmarked against long short-term memory–random forest (LSTM-RF), support vector machine (SVM), 2D-CNN, CNN-LSTM, and residual neural network models. Experimental results show that the proposed algorithm has the highest overall accuracy and Macro-F1 score. It also obtains higher Recall for low-frequency faults. The computational complexity studies indicate that the proposed algorithm has promising potential for real-time satellite health monitoring.

## 1. Introduction

Small satellites have demonstrated strong capabilities in a wide range of space missions, including communications, meteorology, and Earth observation. Although small satellites have a lower cost than traditional large spacecraft [1], they often cannot accommodate dual or triple subsystem redundancies due to volume constraints. As a result, they generally exhibit lower system-level reliability [2]. Typically, data availability and mission success are closely tied to a satellite’s operational reliability [3,4]. Although the system-level reliability of an individual small satellite may be lower, its relatively low manufacturing cost enables its deployment at a constellation scale. Such fleet-level redundancy can partially compensate for the limited redundancy of each individual spacecraft.

Small-satellite constellations have gained increasing popularity over the past decades; for instance, Starlink and OneWeb have deployed thousands of satellites in operation [5]. At the Satellite Technology and Research Centre (STAR), National University of Singapore (NUS), three small satellites are being developed for a formation-flying program [6]. As constellation size grows, operational complexity increases substantially, making manual monitoring and rule-based fault detection progressively inadequate. Continuous health monitoring across a large fleet requires considerable human effort, and delayed detection or isolation of anomalies may interrupt payload operations. In severe cases, faults can propagate and lead to loss of control, resulting in premature mission termination. Therefore, developing reliable and robust fault-diagnosis methods across diverse operating conditions is essential for safeguarding mission success while reducing manpower requirements.

Fault diagnosis has been extensively studied since the late 1970s. It is generally categorized into three groups: model-based methods [7], knowledge-based methods [8], and data-driven methods [9]. Model-based methods construct analytical or physics-informed models to describe system behavior and detect faults by comparing model outputs with measurements. For example, a digital-twin-based approach was proposed in [10] for fault diagnosis and health monitoring of complex satellite systems by integrating physical data with a virtual system. A sliding-mode switching observer was developed to detect and isolate faulty actuators by evaluating structured residual signals [11]. A nonlinear geometric and adaptive-observer-based diagnostic framework for satellite sensors and actuators was proposed in [12]. In this framework, faults are estimated from residuals, while external disturbances and model uncertainties are compensated. Despite their interpretability, model-based methods are highly dependent on model fidelity. As system complexity increases, constructing accurate models becomes increasingly difficult in practice.

To address the limitations of model-based approaches, knowledge-based fault diagnosis methods have been developed to incorporate expert rules and reasoning mechanisms. A rule-based fault diagnosis system was proposed in [13] to interpret alarm messages and localize root causes in the ground segment of satellite communication systems. In addition, fuzzy fault tree analysis was combined with an interpretable interval belief rule base to integrate qualitative expert knowledge with quantitative reliability data for transparent fault diagnosis of aerospace equipment [14]. A hybrid fault detection and severity estimation method that couples an XGBoost-based classifier with rule-based reasoning for complex spacecraft propulsion systems was presented in [15]. These studies suggest that knowledge-based frameworks can provide interpretable decision support for complex aerospace diagnostic tasks. However, constructing comprehensive and maintainable rule bases for large-scale telemetry with heterogeneous signals is often labor-intensive and difficult to generalize across missions and operating conditions.

Since the introduction of the CubeSat standard [16], small satellites have gradually increased in both size and operational complexity, making comprehensive model-based or knowledge-based diagnosis increasingly difficult to develop and maintain. Consequently, data-driven fault diagnosis methods that leverage telemetry data and deep learning (DL) have emerged as a promising solution. DL has demonstrated strong capability for extracting latent patterns from high-dimensional time-series data across multiple domains. For example, the effectiveness of convolutional neural networks (CNNs) for real-time multivariate time-series fault detection in spacecraft attitude sensors was demonstrated in [17], showing that multi-channel CNNs can enhance anomaly-detection performance under complex operating scenarios. A Bayesian LSTM was implemented in [18] to model latent temporal trends in imbalanced satellite telemetry for abnormal event prediction. Furthermore, the short-time Fourier transform was employed in [19] to extract time–frequency features, which were subsequently processed by a deep residual CNN for IMU-related fault identification.

In recent years, DL-based fault diagnosis has advanced rapidly beyond conventional CNN and recurrent architectures. For scarce-label scenarios, a self-supervised train bearing fault diagnosis method based on time–frequency dual-domain prediction (TFDDP) was proposed in [20] to improve representation learning by jointly predicting time-domain and frequency-domain information. In addition, ref. [21] introduced a self-supervised vehicle bearing fault diagnosis method based on time–frequency dual-domain contrast and fusion (TFDDCF) to enhance diagnostic robustness through time–frequency contrastive learning and feature fusion. To improve model adaptability under evolving operating conditions, ref. [22] proposed an incremental machinery fault diagnosis method based on inverted-transformer lifelong learning with a learnable pruning mechanism, enabling continuous task updating while reducing redundant model complexity. Furthermore, a dynamic learnable pruning-based incremental method for low-speed machinery fault diagnosis was introduced in [23] to enhance continual learning capability under newly emerging fault categories or operating conditions.

More computationally expressive deep architectures have also been explored for complex fault-pattern modeling. For example, a deep adversarial capsule network was proposed in [24] for compound fault diagnosis of machinery under multidomain generalization tasks, thereby improving the representation of cross-domain fault features. In addition, ref. [25] proposed a wavelet capsule network (WavCapsNet) with a backward-tracking mechanism for interpretable intelligent compound fault diagnosis. Similarly, ref. [26] introduced a time-series vision transformer (TSViT) for time-series feature modeling in rotating-machinery fault diagnosis. Furthermore, ref. [27] proposed a time–frequency fully connected graph neural network for multisource machine fault diagnosis through multiscale spatiotemporal dependency learning. Collectively, these studies indicate that recent fault diagnosis research is moving toward richer representation learning, stronger adaptability, and improved robustness under realistic data constraints.

However, most existing fault identification methods have primarily been developed for rotating machinery vibration signals or other relatively homogeneous sensing scenarios. In these cases, the inputs often exhibit clearer local fault patterns and more regular sampling behavior. In contrast, satellite telemetry presents a substantially different diagnostic setting. First, telemetry data are highly heterogeneous across subsystems such as EPS, OBC, COMM, and ADCS. Fault-related information is often distributed across multiple subsystems or channels rather than concentrated in a single sensor stream. Second, telemetry signals usually exhibit strong temporal dependencies due to control loops, subsystem interactions, thermal inertia, and mission-state transitions. Therefore, isolated timestamp-level prediction may fail to capture fault evolution. Third, real fault events are extremely scarce compared with abundant healthy observations. This results in a severely long-tailed class distribution and degraded recognition of rare but safety-critical fault modes. Therefore, the key challenge for satellite-telemetry-based fault identification is not merely to employ a more advanced backbone. Instead, it is necessary to construct an appropriate spatiotemporal representation that can expose cross-parameter coupling, preserve short-term fault evolution, and remain effective under severe class imbalance.

Despite advances in DL-based fault diagnosis, most existing methods assume faults and associated telemetry data to be time-independent samples. As a result, they cannot adequately model local fault development dynamics within temporal contexts. In addition, directly feeding high-dimensional raw telemetry vectors into a generic deep model may produce spatially unstructured representations. This makes it difficult to exploit cross-parameter interactions effectively. Furthermore, severe class imbalance between healthy and fault states may bias the learning process toward dominant classes. Consequently, the DL model may become less sensitive to low-frequency, yet mission-critical, faults. These characteristics require a diagnosis framework that jointly considers temporal context modeling, structured multivariate representation, and imbalance-aware learning.

To address the aforementioned challenges, this paper proposes an imbalance-aware spatiotemporal fault diagnosis framework. The framework is based on sliding-window segmentation and a lightweight residual three-dimensional CNN (3D-CNN). First, a sliding-window strategy is introduced to capture short-term fault evolution and local temporal dependencies. These characteristics are difficult to identify from isolated telemetry snapshots. Second, each high-dimensional telemetry vector is reconstructed into a structured two-dimensional (2D) grid according to a predefined mapping. This transformation forms a spatially organized feature representation for learning latent cross-parameter couplings. Consecutive grids are then stacked along the temporal dimension to construct voxel-like spatiotemporal blocks. After this transformation, each sample is represented as a fourth-order tensor. This tensor provides a unified input format for subsequent 3D convolutions and enables joint spatiotemporal modeling. The proposed lightweight residual 3D-CNN then extracts hierarchical spatiotemporal features from these samples and performs end-to-end multi-class classification. To alleviate extreme class imbalance, an effective number-based class-weighting strategy is combined with a class-balanced focal loss function. This design enhances the sensitivity of the model to minority fault categories. Compared with recent diagnosis architectures developed primarily for machinery signals, the proposed framework is specifically tailored for heterogeneous satellite telemetry at the system level. In this setting, diagnostic information is distributed across multiple subsystems and is strongly affected by temporal context and class imbalance. The proposed method is trained and evaluated on the synthetic Lumelite satellite dataset. Furthermore, it is benchmarked against traditional machine-learning methods, sequence-based models, 2D-CNN models, and advanced spatiotemporal DL baselines. These baselines include LSTM-RF [28], SVM [29], 2D-CNN [30], CNN-LSTM [31], ResNet [32], attention-enhanced 3D-CNN with SE module (3D-CNN-SE) [33], TCN-CNN [34], and Transformer [35].

The remainder of this paper is organized as follows. Section 2 introduces the Lumelite series satellite system and the identified fault modes. Section 3 presents the proposed spatiotemporal diagnostic framework and lightweight residual 3D-CNN algorithm. Section 4 reports the experimental results and comparative evaluations. Finally, Section 5 concludes the paper and discusses future work.

## 2. Lumelite Series Satellite

The STAR Centre launched a 12U small satellite, Lumelite-4, in April 2023. Three additional satellites, Lumelite-1 to Lumelite-3, have been developed and qualified for space launch. While Lumelite-4 primarily serves to establish space heritage and demonstrate the VDES payload, Lumelite-1 to Lumelite-3 require continuous monitoring, controlled out-of-plane maneuvering, and precise formation keeping for formation maintenance and intersatellite communication.

The Lumelite series satellites, developed by the STAR Centre, adopt a modular and scalable bus system for small satellites. The bus system supports satellite masses ranging from 12 kg to 70 kg without requiring redesign of the bus electronics. Both the EPS and ADCS can be easily expanded to accommodate higher power or attitude-control precision requirements imposed by the payload [36].

**Figure 1:**
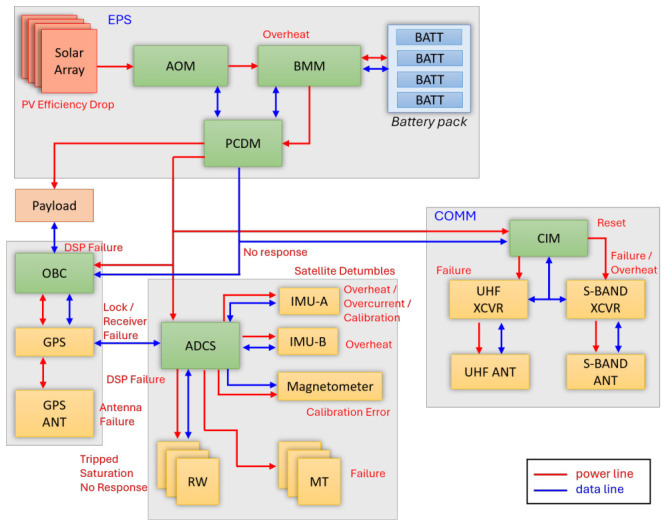
The architecture diagram of Lumelite satellite with a list of identified faults.

The architecture diagram of the Lumelite satellite is shown in Figure 1, where the power lines are shown in red, and the data lines are shown in blue. As shown in Figure 1, the Lumelite satellite bus primarily consists of the EPS, OBC, ADCS, and COMM subsystems. Several faults were identified during the development of the satellite engineering and qualification models. These faults are briefly indicated in Figure 1 and will be discussed in detail in the following subsections. The bus subsystems communicate with each other through an in-house-developed communication protocol based on the CubeSat protocol [37]. For COTS instruments, such as the X-band XCVR and UHF XCVR, communication is conducted using the predefined protocols provided by the manufacturers.

### 2.1. EPS

The three major components of the EPS are the solar array with the AOM, the battery pack with the BMM, and the PCDM. The AOM has five MPPT channels [38] to harvest solar energy from the solar array. For the Lumelite-1 to Lumelite-4 satellites, the solar-cell configuration of each channel consists of four cells in series and one cell in parallel. The AOM then supplies energy to the battery pack through the BMM at an unregulated voltage level. The battery pack contains four series-connected lithium-ion batteries, each with a nominal voltage of 3.6 V and a capacity of 6.8 Ah. The BMM controls the battery charging rate to prevent overvoltage in the battery pack. When the battery pack is fully charged, the BMM allows excess energy to directly power the bus system through the PCDM.

The PCDM is the primary power control and distribution system of the Lumelite satellite system. The supply voltages are 3.3 V, 5 V, and 12 V. The PCDM monitors and controls the electrical energy supplied to the OBC, ADCS, COMM, payload, sensors, and actuators. In addition, the PCDM periodically transmits a request to each subsystem to verify its functionality. If a subsystem fails to acknowledge this request, the PCDM automatically initiates a power-cycling procedure for that subsystem. Furthermore, it collects and stores telemetry data from both the AOM and BMM. These telemetry data are then transmitted to the ground station through the COMM S-band XCVR.

Figure 1 shows that the EPS consists of multiple electronic circuit boards, such as the AOM, BMM, and PCDM. The AOM, BMM, and PCDM operate with low-complexity processes to ensure the stability and reliability of the EPS. Therefore, the MCU temperatures of both the AOM and PCDM are generally stable. However, the BMM is located close to the battery pack, where its MCU temperature may be influenced by the battery temperature. Thus, two potential faults are identified:

- ***PV Efficiency Drop***: The AOM is unable to optimally harvest solar energy from the solar array. This may be caused by defects in the solar array circuit board or by satellite tumbling.
- ***BMM Overheat***: The MCU temperature of the BMM exceeds 80 °C.

### 2.2. OBC

The OBC primarily receives the satellite operation schedule from ground control and operates the payload according to the predefined schedule. In addition, the OBC retrieves mission data from the payload storage when a downlink request is received from ground control. Furthermore, the OBC continuously monitors the GPS receiver status and synchronizes the time information of each subsystem using the updated GPS receiver time.

The OBC on the Lumelite satellite has two DSPs for dual-redundancy. In Figure 1, the GPS receiver and antenna are considered part of the OBC subsystem. Therefore, the identified faults include the following:

- ***OBC DSP Failure***: One of the OBC DSPs fails to operate, and the DSP switching process also fails.
- ***OBC No Response***: The OBC does not respond to the PCDM acknowledgment request.
- ***GPS Lock Failure***: The GPS receiver is unable to lock onto the GPS signal, or it loses lock during operation. As a result, PVT information stops updating.
- ***GPS Failure***: The GPS receiver fails to operate.
- ***GPS Antenna Failure***: The GPS antenna fails to operate and is unable to receive relevant signals.

### 2.3. COMM

The COMM subsystem consists of the CIM, UHF XCVR, and S-band XCVR. The CIM serves as the primary interface module between the S-band/UHF XCVRs and the satellite bus system. Both the S-band XCVR and UHF XCVR have their own communication protocols and data rates. For each TMTC packet sent by a subsystem to the ground station, the CIM converts the TMTC data into the appropriate packet format before forwarding it to either the S-band XCVR or UHF XCVR for transmission.

Because the CIM primarily handles communication between the S-band/UHF XCVRs and other bus subsystems, common faults may occur in the S-band XCVR, UHF XCVR, or CIM:

- ***CIM No Response***: The CIM does not respond to the PCDM acknowledgment request.
- ***CIM Overheat***: The CIM MCU temperature exceeds 50 °C.
- ***UHF Failure***: The UHF XCVR fails to operate.
- ***S-Band Failure***: The S-band XCVR fails to operate.
- ***S-Band Overheat***: The temperature of the S-band XCVR exceeds 55 °C.

### 2.4. ADCS

The Lumelite series satellite is a three-axis-stabilized satellite [39]. The satellite attitude is controlled by RWs, and the momentum stored in the RWs is dumped through MT operation. The ADCS performs three attitude control modes:

1. *Sun pointing*: Primary pointing mode for solar energy harvesting.
2. *Nadir pointing*: Secondary pointing mode for satellite missions.
3. *Target pointing*: Secondary pointing mode for satellite missions.

To achieve nadir and target pointing, the ADCS uses sun sensor and magnetometer measurements to determine the current attitude. It then controls the RWs to orient the satellite toward the desired attitude. Therefore, the ADCS is the most complex bus subsystem. It must communicate with multiple sensors, such as the IMU, sun sensor, GPS receiver, and magnetometer, while controlling actuators such as RWs and MTs. According to [40], each sensor and actuator in the Lumelite satellite communicates through different interfaces and protocols. In addition, each sensor has different data acquisition and response delays. Therefore, the ADCS must handle unsynchronized sensor information while providing precise attitude control.

The functionality of the ADCS is crucial to mission success. Through extensive testing on the satellite engineering model, multiple possible failures were identified. These failures are as follows:

- ***ADCS DSP Failure***: One ADCS DSP fails to operate, and the DSP switching process also fails.
- ***RW Trip***: One RW draws excessive current and is tripped by the PCDM.
- ***RW Saturation***: One RW rotates at its maximum speed, causing the satellite to lose attitude control capability.
- ***RW No Response***: One or multiple RWs do not respond to ADCS commands.
- ***MT Failure***: One or multiple MTs do not respond to ADCS commands.
- ***Magnetometer Calibration Error***: The offset of either IMU-A or the stand-alone magnetometer is incorrectly calibrated. This results in incorrect Earth magnetic field measurements from one of the magnetometers.
- ***IMU-A Overheat***: The IMU-A temperature exceeds 80 °C.
- ***IMU-B Overheat***: The IMU-B temperature exceeds 80 °C.
- ***IMU-A Overcurrent***: IMU-A draws excessive current, resulting in a temperature rise.
- ***IMU-A Gyroscope Calibration Error***: Incorrect gyroscope bias calibration causes a constant bias offset in pointing accuracy.
- ***ADCS Tumble***: The satellite rotates uncontrollably at more than 3 °/s.

In summary, possible common failures were identified within each bus subsystem. However, a satellite system contains thousands of parameters, and not all parameters are useful or correlated with the identified faults. Therefore, a total of 221 telemetry parameters across the EPS, OBC, CIM, and ADCS are selected.

### 2.5. Fault Data Simulation

Although the faults in Section 2.1, Section 2.2, Section 2.3 and Section 2.4 were identified through extensive testing on the Lumelite series satellite engineering model, their natural occurrence remains rare. To ensure that all representative fault categories are sufficiently observed by the proposed algorithm during training and testing, a satellite system simulator is constructed to reproduce these faults. It should be emphasized that this process does not produce a balanced dataset. Instead, the generated telemetry retains a realistic long-tailed distribution dominated by the healthy state while increasing the representation of rare fault categories compared with their infrequent occurrence in practice. The simulated telemetry parameters and their corresponding fault labels are provided in the dataset.

**Figure 2:**
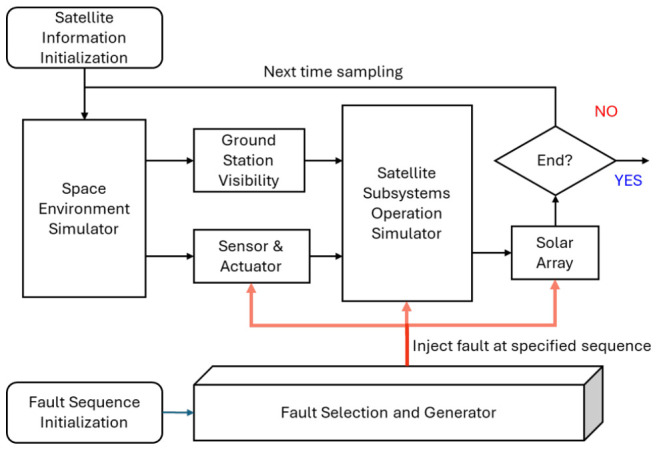
Process flow of the satellite system simulator with fault generation features.

The satellite system simulator flow diagram is presented in Figure 2. The Lumelite-4 two-line element data [41] from Space-Track are used to simulate the satellite orbit. In addition, the Lumelite-4 engineering-model test data collected over a three-month period and the corresponding flight-model in-orbit data are used as references to configure the nominal operating parameters of the simulated satellite system.

The simulator consists of three parts: The space environment simulator, the satellite subsystem operation simulator, and the fault selection and generation module. The space environment simulator provides satellite time, position, and velocity information. It also provides the sun’s direction, the sunlight or eclipse condition, and Earth’s magnetic field readings experienced by the satellite. This information is processed to simulate sensor readings and actuator operations. Furthermore, the NUS ground station is considered for ground-contact visibility analysis, which is essential for S-band and UHF XCVR operations.

The satellite subsystem operation simulator includes the detailed operation of each component within each satellite subsystem. The operating parameters are categorized into non-configurable and configurable parameters. The non-configurable parameters include the voltage and current levels of each component, selected component temperatures, GPS receiver outputs, sensor outputs, reaction wheel responses, microcontroller total and session uptimes, and other related parameters. It should be noted that different subsystems may exhibit changes in current draw and temperature under different operating conditions. For example, the battery pack experiences high current draw during eclipse periods because the solar array is unable to generate charging energy.

The configurable parameters include the RW set speed, attitude control mode, attitude control duration, RW and MT operation modes, OBC and ADCS DSP selection, and related settings. The satellite fault scenario sequence is constructed by the fault selection and generation module. All faults in Section 2.1, Section 2.2, Section 2.3 and Section 2.4 are randomly sequenced and repeated for a preset number of cycles. The duration of each fault occurrence is randomly selected within a predefined range. When a fault occurs, the corresponding abnormal parameters are injected into the simulator to replace the nominal operating parameters.

Solar energy generation is simulated based on the sunlight condition and satellite subsystem operating conditions. The generated energy charges or discharges the battery pack, depending on the operation scenario.

## 3. Proposed Methodology

**Figure 3:**
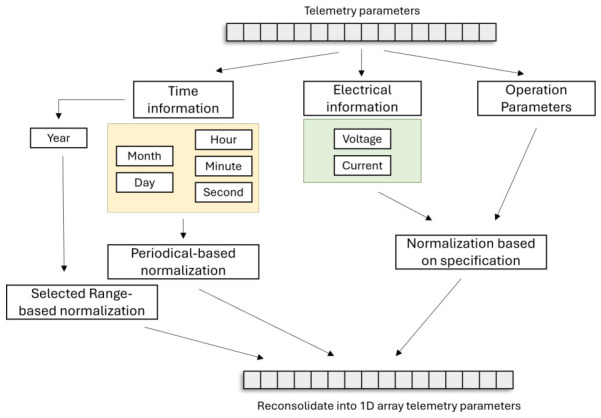
Telemetry parameter preprocessing.

This section presents the proposed spatiotemporal fault diagnosis framework. The telemetry transmitted by each subsystem contains various data types, such as string, binary, and numerical values. In addition, each component, sensor, actuator, and payload has different operating parameters and conditions. Therefore, these parameters need to be scaled or normalized before they are reconstructed into a 2D grid to form spatial feature maps. Three normalization methods are considered for satellite telemetry data, as shown in Figure 3:

- **Periodical-based normalization:** The month, day, hour, minute, and second in each telemetry sample transmitted by each subsystem are normalized into sine or cosine representations.
- **Range-based normalization:** The year information in each telemetry sample transmitted by each subsystem is normalized with respect to a selected year range, typically from the launch year to the end-of-life year.
- **Specification-based normalization:** Electrical information, such as voltage and current, and other operating parameters, such as temperature, are normalized based on their respective operational ranges.

The normalized telemetry samples are then fed into the proposed framework.

### 3.1. Framework Overview

Let $x_{t}\in R^{N}$ denote the multivariate telemetry vector at time index *t*, where *N* is the number of monitored parameters across multiple subsystems, such as EPS, OBC, ADCS, and COMM. Given a telemetry stream ${\left\{x_{t}\right\}}_{t=1}^{T}$, the goal of system-level fault diagnosis is to infer the operational state

(1) $$y_{t}\in \left\{1,2,\ldots ,K\right\},$$

where each class corresponds to a specific fault mode or the healthy condition.

**Figure 4:**
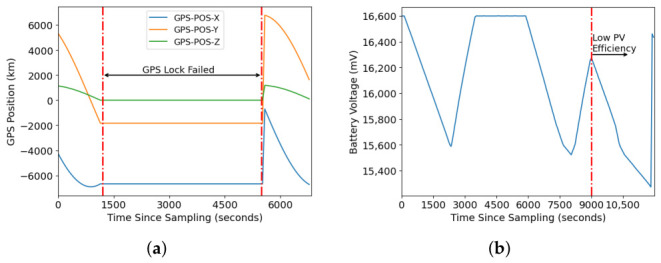
Examples of faults occurring within the satellite system over time: (**a**) GPS Lock Failure and (**b**) PV Efficiency Drops.

Satellite faults often manifest as progressive and coupled deviations rather than instantaneous outliers. For example, Figure 4a illustrates that when a GPS receiver fails to maintain continuous lock on GPS signals, the last known position information is not updated until the receiver reacquires and decodes the signals. Similarly, Figure 4b shows that reduced solar energy harvesting efficiency may not be immediately reflected in the battery voltage profile. In this case, the battery profile may be misinterpreted as an eclipse condition.

**Figure 5:**
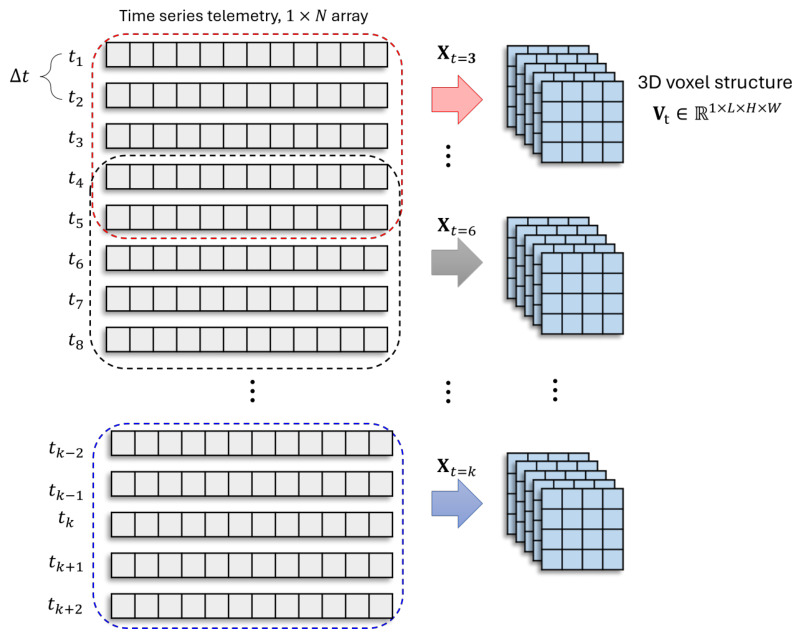
Example of reconstruction of telemetry data into a temporal-based format with L=5.

**Figure 6:**
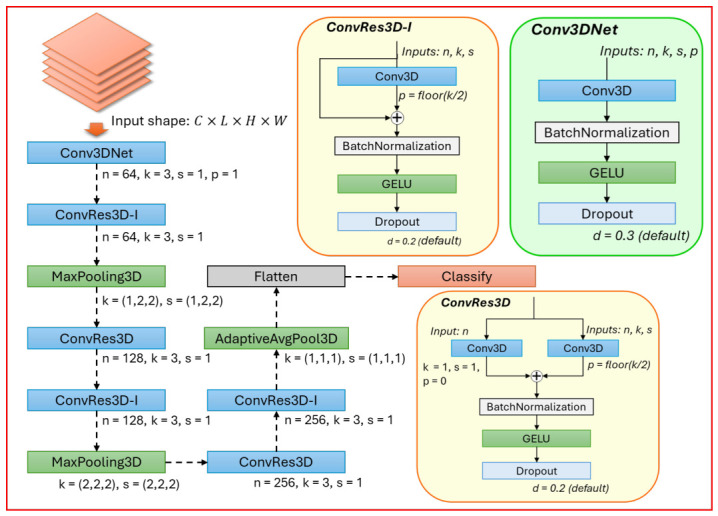
The architecture of the proposed 3D CNN-Resnet.

These fault characteristics motivate the use of a spatiotemporal representation learning approach that (i) captures local temporal evolution, (ii) exposes latent inter-parameter dependencies through structured embedding, and (iii) performs joint temporal–spatial feature extraction. Accordingly, we propose a three-stage framework:

1. **Temporal Context Modeling via Sliding Windows**: a sliding-window operator constructs local telemetry segments to capture short-term evolution patterns, as shown in Figure 5.
2. **Structured Spatial Embedding of Telemetry Features**: each high-dimensional telemetry vector is mapped to a 2D grid to introduce a convolution-friendly inductive bias for learning parameter couplings, as shown in Figure 5.
3. **Spatiotemporal Classification with Residual 3D-CNN**: a lightweight residual 3D-CNN extracts hierarchical spatiotemporal features and outputs the fault category, as shown in Figure 6.

### 3.2. Temporal Context Modeling via Sliding Windows

Telemetry signals typically exhibit temporal dependencies due to control loops, thermal inertia, sensor delays, and subsystem interactions. Therefore, point-wise diagnosis based on a single timestamp may overlook early fault signatures. To explicitly incorporate local temporal context, we apply a fixed-length sliding window with size *L* and stride *s* to the telemetry stream.

For each center time *t*, let

(2) $$r_{-}=\left\lfloor \frac{L}{2}\right\rfloor ,\;r_{+}=\left\lceil \frac{L}{2}\right\rceil -1,$$

where ⌊·⌋ and ⌈·⌉ denote the floor and ceiling operations, respectively. The windowed temporal segment is then constructed as

(3) $$X_{t}={\left[x_{t-r_{-}},\;x_{t-r_{-}+1},\;\ldots ,\;x_{t},\;\ldots ,\;x_{t+r_{+}}\right]}^{⊤}\in R^{L\times N}.$$

Equations (2) and (3) ensure that each temporal segment contains exactly *L* telemetry samples. When *L* is odd, the window is symmetric around the center time *t*. In this paper, L=5. Thus, the window covers two samples before and two after the center time, i.e., $\left\{x_{t-2},x_{t-1},x_{t},x_{t+1},x_{t+2}\right\}$. It is noted that the floor and ceiling operations are used only to determine integer window boundaries. No rounding is applied to the telemetry values.

The window-level label is assigned as the class at the window center, i.e., $y_{t}$ defined in Equation (1). This strategy enables the model to learn local fault progression patterns and short-term temporal dependencies. It also maintains a clear correspondence between each temporal segment and its diagnostic label. In this study, this formulation corresponds to a single-label multi-class diagnosis setting. Each temporal segment is associated with one dominant operational state rather than multiple concurrent fault labels.

### 3.3. Structured Spatial Embedding of Telemetry Features

Although each $x_{t}$ in Equation (3) is a high-dimensional telemetry vector, it lacks explicit spatial structure. Applying convolution directly to a one-dimensional vector may not effectively exploit local interactions among functionally related telemetry channels. Therefore, a structured 2D embedding is introduced to provide a convolution-friendly input representation for subsequent spatiotemporal feature extraction.

In this study, the 2D arrangement is not a purely arbitrary reshape operation. It is also not intended to exactly reproduce the physical topology or causal graph of the satellite bus. Instead, a deterministic subsystem and function-informed mapping is adopted. Specifically, telemetry parameters are first grouped according to their associated satellite subsystems, including EPS, OBC, COMM/CIM, and ADCS. Within each subsystem group, parameters are further arranged according to their functional or signal categories, such as electrical measurements, thermal measurements, sensor outputs, actuator status, communication/status indicators, and control-related variables. In this way, telemetry channels that are functionally related or generated from the same subsystem are placed in neighboring or nearby regions of the 2D grid. This provides a weak but meaningful spatial inductive bias for the 3D-CNN. It allows local convolutional kernels to capture short-range cross-parameter interactions within subsystem- or function-level neighborhoods.

Let π(·) denote the predefined telemetry ordering after subsystem and function-based grouping. Each telemetry parameter $x_{t,i}$ is assigned to a corresponding grid position according to this deterministic mapping. The resulting 2D representation is denoted as

(4) $$G_{t}={\mathrm{Map}}_{\pi }\left(x_{t}\right)\in R^{H\times W}.$$

When the number of telemetry parameters satisfies N<HW, zero-padding is appended only after all valid telemetry channels have been placed in the grid. When N>HW, truncation can be applied as a general implementation option. However, in this study, 221 telemetry parameters are used, and the selected grid size is 40×40. Therefore, no feature truncation occurs in the reported experiments. The transformed grids within a temporal window are then stacked along the temporal dimension to form a voxel-like tensor:

(5) $$V_{t}\in R^{1\times L\times H\times W}.$$

The resulting tensor $V_{t}$ serves as the input to the 3D convolutional classifier. This enables the network to jointly model temporal continuity along the window dimension and local cross-parameter interactions within the structured telemetry grid.

It should be clarified that the telemetry-to-grid mapping does not change the number of convolutional filters in the CNN. The number of filters is determined by the network architecture, namely the predefined output channels of each convolutional layer. The reshape operation only changes the spatial organization and resolution of the input representation. Therefore, it affects computational efficiency indirectly through the grid size H×W, rather than directly changing the number of learnable filters.

For a 3D convolutional layer, the number of learnable parameters is mainly determined by the kernel size, input channels, and output channels. In contrast, FLOPs and memory usage are related to the number of output feature-map positions, which depends on the input dimensions *L*, *H*, and *W*. Therefore, a larger grid size H×W increases the number of convolutional operations and memory usage, even though it does not increase the number of filters. In this study, the selected 221 telemetry parameters are mapped into a 40×40 grid. Since 221<40×40, zero-padding is appended after all valid telemetry channels, and no telemetry parameter is truncated in the reported experiments.

### 3.4. Spatiotemporal Classification with Residual 3D-CNN

To clarify the difference between the proposed residual 3D-CNN and 2D-CNN baseline, both models use the same telemetry-to-grid embedding strategy, but differ in how temporal information is represented and processed. The 2D-CNN baseline takes a single telemetry grid as input, i.e., $G_{t}\in R^{1\times H\times W}$, and applies 2D convolutional kernels only over spatial dimensions. Therefore, it can learn local cross-parameter patterns within a single telemetry snapshot but cannot explicitly model how these patterns evolve over time.

In contrast, the proposed residual 3D-CNN stacks consecutive telemetry grids within a sliding window to form a voxel-like tensor $V_{t}\in R^{1\times L\times H\times W}$. The 3D convolutional kernels, with the form $k_{L}\times k_{H}\times k_{W}$, operate jointly along the temporal and spatial dimensions. In this way, the proposed model extends the receptive field from a static spatial grid to a local spatiotemporal block. The pooling operation is also performed over the learned spatiotemporal feature volume rather than over a single 2D feature map. Therefore, the proposed architecture is designed to capture both subsystem-level cross-parameter interactions and their short-term temporal evolution.

Given the voxelized input $V_{t}$, a lightweight residual 3D-CNN is proposed to learns hierarchical spatiotemporal features from the local telemetry block. Based on this representation, the 3D convolutional layers extract evolving coupling patterns across telemetry parameters within each sliding window. The architecture of the proposed lightweight residual 3D-CNN is presented in Figure 6. Here, *n* denotes the number of filters; *k* denotes the kernel size; *p* denotes the zero-padding size; and *s* denotes the stride size.

Let $V\in R^{C_{\mathrm{in}}\times L_{\mathrm{in}}\times H_{\mathrm{in}}\times W_{\mathrm{in}}}$ denote an intermediate feature volume, where $L_{\mathrm{in}}$ is the temporal sliding-window length and $\left(H_{\mathrm{in}},W_{\mathrm{in}}\right)$ are the spatial dimensions of the embedded 2D grid. A 3D convolution computes the output feature map $Y\in R^{C_{\mathrm{out}}\times L_{\mathrm{out}}\times H_{\mathrm{out}}\times W_{\mathrm{out}}}$ as

(6) $$Y_{c_{\mathrm{out}}}^{\left(t,h,w\right)}=\sum_{c_{\mathrm{in}}=1}^{C_{\mathrm{in}}}\sum_{\tau =0}^{k_{L}-1}\sum_{i=0}^{k_{H}-1}\sum_{j=0}^{k_{W}-1}\Theta_{c_{\mathrm{out}},c_{\mathrm{in}}}^{\left(\tau ,i,j\right)}\;V_{c_{\mathrm{in}}}^{\left(t+\tau ,\;h+i,\;w+j\right)}+b_{c_{\mathrm{out}}},\;c_{\mathrm{out}}=1,\ldots ,C_{\mathrm{out}}.$$

where (t,h,w) indexes the output location, and $\left(k_{L},k_{H},k_{W}\right)$ denotes the 3D kernel size along the temporal and spatial axes of the 2D grid, as shown in Figure 5 and Figure 6. Here, $\Theta_{c_{\mathrm{out}},c_{\mathrm{in}}}^{\left(\tau ,i,j\right)}$ is the learnable kernel weight at offset (τ,i,j) connecting the $c_{\mathrm{in}}$-th input channel to the $c_{\mathrm{out}}$-th output channel, and $b_{c_{\mathrm{out}}}$ is the corresponding bias term. $V_{c_{\mathrm{in}}}^{\left(t+\tau ,\;h+i,\;w+j\right)}$ denotes the input element of V sampled at the $c_{\mathrm{in}}$-th channel and location (t+τ,h+i,w+j) covered by the 3D kernel. Meanwhile, $Y_{c_{\mathrm{out}}}^{\left(t,h,w\right)}$ represents the response of Y at the output location (t,h,w). Note that the temporal kernel size $k_{L}$ is applied *within* a window of length $L_{\mathrm{in}}$, typically with $k_{L}\leq L_{\mathrm{in}}$.

To mitigate gradient degradation and stabilize deep feature learning, residual blocks are adopted, as shown by **ConvRes3D-I** and **ConvRes3D** in Figure 6. For an input feature volume U, the residual output Res(U) is defined as

(7) Res(U)=U+F(U),

where F(·) is a nonlinear transformation composed of 3D convolution, normalization, and activation. ReLU is widely used but may suffer from vanishing-gradient and neuron-death problems [42]. To mitigate these issues, GELU is adopted as the primary activation function for all layers except the classification layer, as shown in Figure 6 [43].

After multiple residual stages with downsampling, adaptive global average pooling is applied over the spatiotemporal dimensions. This operation compresses the final feature maps into a compact channel-wise descriptor:

(8) $$z_{c_{out}}=\frac{1}{L_{out}H_{out}W_{out}}\sum_{t=1}^{L_{out}}\sum_{h=1}^{H_{out}}\sum_{w=1}^{W_{out}}F_{c_{out}}^{\left(t,h,w\right)},\;c_{out}=1,\ldots ,C_{out},$$

where F is the last convolutional output and $z\in R^{C}$ is the pooled feature vector. The classifier predicts the fault category using a fully connected layer and softmax function [44]:

(9) $${\hat{p}}_{k}=\frac{\exp({\hat{w}}_{k})}{\sum_{j=1}^{K}\exp\left({\hat{w}}_{j}\right)},\;k=1,\ldots ,K,$$

where $\hat{w}=Wz+b$. Here, $W\in R^{K\times C_{\mathrm{out}}}$ and $b\in R^{K}$ are learnable parameters of the fully connected classifier, and $\hat{w}={\left[{\hat{w}}_{1},\ldots ,{\hat{w}}_{K}\right]}^{⊤}$ denotes the logit vector before normalization. The softmax function converts logits into a categorical probability distribution $\hat{p}={\left[{\hat{p}}_{1},\ldots ,{\hat{p}}_{K}\right]}^{⊤}\in R^{K}$, satisfying ${\hat{p}}_{k}\in \left(0,1\right)$ and $\sum_{k=1}^{K}{\hat{p}}_{k}=1$. The predicted fault category for center time *t* is obtained by

(10) $${\hat{y}}_{t,k}=\arg\max_{k}{\hat{p}}_{k}$$

### 3.5. Imbalance-Aware Objective: Class-Balanced Focal Loss

Satellite telemetry datasets are typically highly imbalanced: healthy samples dominate, while safety-critical faults may be rare. Optimizing with standard cross-entropy often biases the classifier toward the majority class. To prioritize rare-fault recall, we adopt an effective-number-based class re-weighting strategy [45] and focal modulation [46]. We also follow representative long-tailed learning practices that emphasize minority-class generalization [47,48]. Specifically, the former accounts for the diminishing marginal benefit of added data, while the latter down-weights easy examples and focuses the model on hard-to-classify minority faults.

For class *k* with sample count $n_{k}$, the effective number of samples is defined as [45]

(11) $$E\left(n_{k}\right)=\frac{1-\beta^{n_{k}}}{1-\beta },\;\beta \in \left[0,1\right),$$

and the normalized class weight is

(12) $$\alpha_{k}=\frac{1/E(n_{k})}{\sum_{j=1}^{K}1/E\left(n_{j}\right)}.$$

Given a training sample with the ground-truth *k*-th class and corresponding predicted probability ${\hat{p}}_{k}$ in Equation (9), the class-balanced focal (CB-Focal) loss is defined as [45,46]

(13) $$L_{\text{CB-}\mathrm{Focal}}=\frac{1}{B}\sum_{i=1}^{B}\sum_{k=1}^{K}-y_{k}^{\left(i\right)}\alpha_{k}{\left(1-{\hat{p}}_{k}^{\left(i\right)}\right)}^{\gamma }\log\left({\hat{p}}_{k}^{\left(i\right)}\right),\;\gamma >0,$$

where *B* denotes the mini-batch size, and *K* is the total number of classes. The term $y_{k}^{\left(i\right)}\in \left\{0,1\right\}$ represents the one-hot encoded ground-truth label for the *i*-th sample, and ${\hat{p}}_{k}^{\left(i\right)}$ is the predicted probability of class *k* obtained from the softmax function. The modulating factor ${\left(1-{\hat{p}}_{k}^{\left(i\right)}\right)}^{\gamma }$ down-weights well-classified samples and focuses training on hard-to-classify examples. The weighting factor $\alpha_{k}$ compensates for class imbalance by assigning larger weights to minority classes. The overall objective minimizes the average CB-Focal loss over all samples within a mini-batch.

The proposed training pipeline is summarized in Algorithm 1. For each mini-batch, the sliding-window operation in Equation (3) constructs temporal segments $X_{t}$. Next, each $x_{t}$ is embedded into a 2D grid $G_{t}\in R^{H\times W}$ and stacked into $V_{t}$ using Equation (5). The predicted probability $\hat{p}$ is then computed for each $V_{t}$. Afterward, the class-balanced focal loss $L_{\text{CB-}\mathrm{Focal}}$ is calculated using each predicted output and ground-truth label pair. Finally, Θ and b are updated through gradient-based optimization. This process is repeated for each mini-batch and epoch until training is completed.
**Algorithm 1** Training pipeline of the proposed spatiotemporal fault diagnosis framework.**Require:** Telemetry stream ${\left\{x_{t},y_{t}\right\}}_{t=1}^{T}$; window size *L*; grid size (H,W); network parameters Θ and b.1:**for** e=1 to *E* **do**2:    **for** each mini-batch B **do**3:        Construct temporal segments $X_{t}$ using sliding windows (Equation (3)).4:        Embed each $x_{t}$ into a 2D grid $G_{t}\in R^{H\times W}$ and stack into $V_{t}$ (Equation (5)).5:        Forward pass: $\hat{p}\leftarrow f_{\Theta }\left(V_{t}\right)$ (Equation (9)).6:        Compute class-balanced focal loss $L_{\text{CB-}\mathrm{Focal}}$ (Equation (13)).7:        Given $L_{\text{CB-}\mathrm{Focal}}$, update Θ and b using gradient-based optimization.8:    **end for**9:**end for**

## 4. Experimental Verification and Discussion

### 4.1. Experimental Setup

The proposed method is benchmarked against LSTM–RF, SVM, 2D-CNN, CNN-LSTM, ResNet, attention-enhanced 3D-CNN with SE module (3D-CNN-SE), TCN-CNN, and Transformer in terms of Accuracy, Precision, Recall, and F1-score [49]. All experiments were conducted on Dell Precision 7810 workstation (Dell Inc. Round Rock, TX, USA) equipped with an Intel(R) Xeon(R) CPU E5-2687W v4 @ 3.00 GHz (Intel Corporation, Santa Clara, CA, USA), 4 GB RAM, and an NVIDIA Quadro M5000 GPU (NVIDIA Corporation, Santa Clara, CA, USA). The proposed model was implemented in Python 3.8 (Python Software Foundation, Wilmington, DE, USA) using the PyTorch 2.3.1 deep learning framework (PyTorch Foundation, Linux Foundation, San Francisco, CA, USA), with CUDA 11.8 (NVIDIA Corporation, Santa Clara, CA, USA) acceleration enabled on the NVIDIA Quadro M5000 GPU.

The experiments were performed on the Lumelite satellite telemetry dataset, which contains one healthy class and 23 representative fault types identified from the Lumelite satellite engineering-model inspection. These faults cover the main bus subsystems, as presented in Section 2. Specifically, the modeled fault types include PV efficiency drop, BMM overheat, OBC DSP failure, OBC no response, GPS lock failure, GPS failure, GPS antenna failure, CIM no response, CIM overheat, UHF failure, S-band failure, S-band overheat, ADCS DSP failure, reaction wheel trip, reaction wheel saturation, reaction wheel no response, magnetic torquer failure, magnetometer calibration error, IMU-A overheat, IMU-B overheat, IMU-A overcurrent, IMU-A gyroscope calibration error, and ADCS tumble.

Two datasets were created for training and testing purposes:

- ***Train Dataset***: starting from 22 April 2024, with a total duration of 60 days and a sampling interval of 60 s.
- ***Test Dataset***: starting from 15 March 2025, with a total duration of 36 days and a sampling interval of 60 s.

As presented in Section 2.5, the temporal sequence of fault events was generated by randomly permuting the predefined fault types and injecting them one at a time into the telemetry sequence until the end of the simulation period. Therefore, the **random sequence** in this paper refers to the randomized temporal ordering of predefined fault events, rather than undefined fault categories or simultaneous multi-fault occurrences. Different faults do not simultaneously occur. The current benchmark follows a single-fault labeling protocol, where each window is assigned one dominant operational-state label. For each injected fault event, the duration was randomly selected within the range of 40 to 90 min. This range was selected based on three considerations. First, the telemetry sampling interval is 60 s, and the adopted sliding-window length is L=5, corresponding to a 5-min local temporal context. Therefore, a 40 to 90 min duration provides sufficient telemetry samples and sliding windows for learning local fault-evolution patterns. Second, this duration range corresponds to short-to-moderate operational fault episodes rather than to unrealistically long continuous failures. This prevents fault segments from dominating the dataset. Third, it helps ensure that all representative fault types appear in both the training and test sets while preserving a long-tailed and imbalanced distribution dominated by the healthy state.

Although the simulator increases the occurrence of representative fault events, the resulting datasets are not artificially balanced. The telemetry generation process was designed to preserve a realistic long-tailed operational distribution dominated by the healthy state. This process is referred to as fault-event enrichment rather than class balancing.

**Figure 7:**
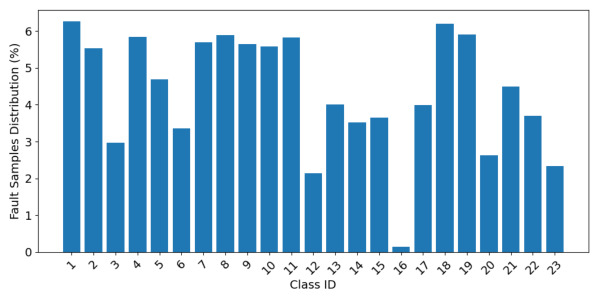
Faults distribution in test dataset.

Figure 7 shows that the healthy class contains more than 24,000 samples in the test dataset. Most fault classes contain between 800 and 1700 samples, reflecting the long-tailed imbalance commonly observed in satellite health-monitoring scenarios. In contrast, the MT failure contains only 36 samples. MTs primarily operate under sunlight conditions for RW momentum dumping. This process is activated only when the RW speed exceeds a specific threshold. The limited operating windows make MT failure occurrences rare. Therefore, the generated training and testing datasets remain strongly imbalanced.

### 4.2. Configuration of Proposed Algorithm

Before model training, several preprocessing and input-construction steps were applied to enable spatiotemporal modeling of telemetry signals. Each *N*-dimensional telemetry vector was mapped into a 40×40 two-dimensional grid according to the subsystem- and function-informed ordering described in Section 3.3. Since the number of selected telemetry parameters is 221, which is smaller than the grid capacity of 40×40=1600, zero-padding was appended to all valid telemetry channels. No telemetry parameter was truncated in the reported experiments. The resulting voxel volume has a shape of 1×L×H×W and serves as the input to the 3D-CNN classifier.

However, the sliding-window length *L* in (2) and the CB-Focal loss parameters β and γ in Equation (13) must be carefully selected, as they may affect model performance. Therefore, Table 1 presents the sensitivity analysis of fault diagnosis performance with respect to *L*, β, and γ. The value of *L* was varied to examine the effect of temporal context on fault-evolution modeling, while β and γ were adjusted to evaluate the trade-off between minority-class emphasis and optimization stability.

Table 1 shows that diagnostic performance is influenced by *L*, β, and γ. Both Recall and F1-score are lower when L=3, indicating that a short temporal window may be insufficient to capture local fault-evolution patterns. The overall performance improves when L=5, suggesting that moderate temporal context is beneficial for satellite telemetry fault diagnosis. However, further increasing the window length to L=7 does not lead to consistent improvement. This may be because longer windows introduce redundant information or mixed operational states near fault-transition boundaries.

The parameters β and γ also affect the trade-off between minority-class sensitivity and overall classification stability. A smaller value of β=0.99 weakens the effective-number-based class re-weighting and results in lower Recall. In contrast, a larger value of β=0.9999 improves Recall but decreases Accuracy and Precision, indicating that excessive minority-class emphasis may compromise the recognition stability of majority or easily confused classes. Similarly, increasing γ from 1.0 to 2.0 improves the balance between hard-sample learning and stable optimization, whereas γ=3.0 further increases Recall at the cost of reduced Accuracy and Precision. Overall, L=5, β=0.999, and γ=2.0 achieve the highest Accuracy and Precision while maintaining competitive Recall and F1-score. Therefore, this configuration is selected as the final setting because it provides the best overall trade-off between rare-fault sensitivity and classification stability.

β and γ both affect the trade-off between minority-class sensitivity and classification stability. When β=0.99, effective-number-based class re-weighting is weakened, resulting in lower Recall. When β=0.9999, Recall improves, but Accuracy and Precision decrease. This indicates that excessive minority-class emphasis may compromise majority-class or easily confused class recognition. Similarly, increasing γ from 1.0 to 2.0 improves the balance between hard-sample learning and stable optimization. Although γ=3.0 further increases Recall, it reduces Accuracy and Precision.

The detailed hyperparameter configuration used in the experiments is summarized in Table 2. Overall, L=5, β=0.999, and γ=2.0 are selected because this configuration achieves the highest Accuracy and Precision while maintaining competitive Recall and F1-score. This combination provides the best trade-off between rare-fault sensitivity and classification stability. The same preprocessing pipeline and identical train/validation/test splits were used for all baseline models to ensure a fair and reproducible comparison.

### 4.3. Classification Performance Evaluation

To evaluate the effectiveness of the proposed model for small-satellite fault diagnosis, four widely used classification metrics are adopted: Accuracy, Macro-Precision, Macro-Recall, and Macro-F1 score [49]. These metrics capture complementary aspects of model performance and are suitable for scenarios with multiple fault categories and highly imbalanced sample distributions.

Let *K* denote the total number of classes, and let $TP_{k}$, $FP_{k}$, and $FN_{k}$ represent the true positives, false positives, and false negatives for class *k*, respectively. The evaluation metrics are defined as follows:

(14) $$\mathrm{Accuracy}=\frac{\sum_{k=1}^{K}TP_{k}}{\sum_{k=1}^{K}N_{k}}$$

(15) $$\text{Macro-}\mathrm{Precision}=\frac{1}{K}\sum_{k=1}^{K}\frac{TP_{k}}{TP_{k}+FP_{k}}$$

(16) $$\text{Macro-}\mathrm{Recall}=\frac{1}{K}\sum_{k=1}^{K}\frac{TP_{k}}{TP_{k}+FN_{k}}$$

(17) $$\text{Macro-}F1=\frac{1}{K}\sum_{k=1}^{K}\frac{2\;\cdot \;{\mathrm{Precision}}_{k}\;\cdot \;{\mathrm{Recall}}_{k}}{{\mathrm{Precision}}_{k}+{\mathrm{Recall}}_{k}}$$

Table 3 shows that the proposed model achieves the best performance across all evaluation metrics, demonstrating its superior diagnostic capability for imbalanced satellite telemetry fault diagnosis.

Traditional machine-learning models, such as SVM and LSTM–RF, show relatively limited performance because they rely heavily on manually engineered features. They also cannot sufficiently capture the temporal dependencies and cross-parameter interactions in satellite telemetry. Sequence-based models, such as CNN-LSTM and TCN-CNN, can model temporal dependencies to some extent. However, their spatial and temporal feature extraction processes are usually separated or only weakly coupled. Similarly, 2D-CNN and ResNet can learn spatial patterns from telemetry-grid representations, but they do not explicitly model fault evolution across consecutive telemetry windows.

In contrast, the proposed residual 3D-CNN applies convolution over voxelized telemetry windows and jointly exploits spatial and temporal dimensions. This design enables the model to capture local cross-parameter interactions and short-term fault-evolution patterns within a unified spatiotemporal feature-learning framework.

As shown in Table 3, compared with the 2D-CNN, the proposed residual 3D-CNN improves Accuracy from 78.23% to 94.62%, Precision from 80.51% to 96.45%, Recall from 73.84% to 90.57%, and F1-score from 77.00% to 93.00%. Both the proposed residual 3D-CNN and the 2D-CNN use the same telemetry-to-grid embedding strategy. The improvements indicate that the performance gain is not only due to the structured telemetry-grid representation, but also to the explicit modeling of short-term temporal fault evolution. This is particularly important for satellite telemetry, where faults often develop gradually and may only become distinguishable through temporal changes across multiple subsystem parameters.

In addition, the proposed model outperforms advanced spatiotemporal baselines, including 3D-CNN-SE, TCN-CNN, and Transformer. For example, compared with 3D-CNN-SE, the proposed model improves Accuracy, Recall, and F1-score by 4.77, 5.35, and 4.85 percentage points, respectively. These results indicate that the proposed residual 3D-CNN provides more effective spatiotemporal representation learning for both frequent and rare satellite fault categories.

To further investigate the class-level discriminative capability of different methods, Table 4 reports the class-wise recall values for all 24 categories on the test set.

For relatively easier classes, such as Classes 7, 8, 11, 12, and 19, most methods achieve high Recall values, while the proposed method maintains stable and leading performance. These results indicate that the proposed method improves both overall diagnostic performance and class-wise robustness. Future improvements for low-recall rare classes may include rare-fault data augmentation, physics-guided fault injection, class-specific threshold calibration, hierarchical diagnosis, and temporal transition modeling.

It should be noted that several rare classes remain difficult to recognize. Table 4 shows that several comparison methods yield near-zero Recall for Classes 4, 10, 21, and 23, whereas the proposed method attains 97.4%, 55.5%, 84.4%, and 60.9%, respectively. The relatively low Recall of some rare classes may be attributed to three factors. First, the number of available samples for these categories is very limited, which restricts the intra-class variability learned by the model. Second, some rare faults may produce weak or indirect telemetry responses, where fault signatures are distributed across multiple subsystem parameters rather than concentrated in a few dominant channels. Third, these classes may share similar telemetry patterns with normal operating states or other fault categories, especially when the fault effect is influenced by sunlight/eclipse conditions, mission mode, or subsystem operating status.

The high recall achieved by the proposed method for Classes 4 and 21 suggests that the proposed spatiotemporal representation and CB-Focal loss improve sensitivity to minority fault modes. However, the lower Recall values for Classes 10 and 23 indicate that extremely rare fault modes cannot be fully resolved by loss re-weighting and spatiotemporal feature learning alone, even though the proposed imbalance-aware 3D-CNN improves minority-class recognition compared with most baselines. This challenge is mainly due to the combined effects of limited samples, weak telemetry signatures, and high feature similarity with other operating states, rather than a failure of the overall spatiotemporal diagnostic framework. Therefore, further optimization is still required for extremely rare classes such as Classes 10 and 23.

Overall, the proposed method achieves superior or highly competitive performance on most classes and shows clear advantages on several challenging minority categories.

### 4.4. Feature Visualization

**Figure 8:**
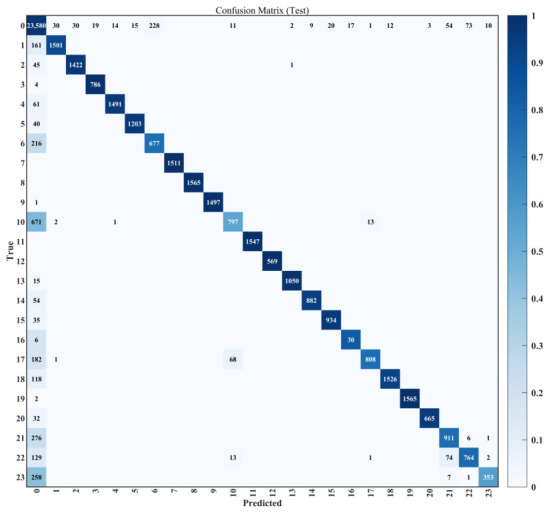
Confusion matrix of the proposed model.

Figure 8 illustrates the confusion matrix of the proposed model, providing a detailed assessment of the classification performance for all 23 fault modes and one healthy mode. Figure 8 shows that most categories exhibit strong diagonal dominance, indicating that the model can accurately discriminate most fault types.

**Figure 9:**
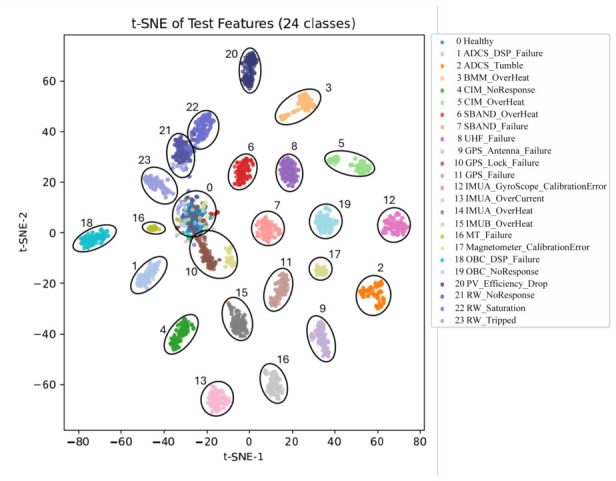
t-SNE visualization of the proposed model.

To further analyze the learned representations, t-SNE projection [50] was applied to the high-level feature embeddings extracted from the final convolutional layer. Figure 9 shows that each fault class forms a compact and well-isolated cluster in the latent space, confirming that the network successfully learns intrinsic fault manifolds embedded within the telemetry data. However, Figure 9 shows that the algorithm occasionally fails to detect satellite anomalies and mistakenly identifies them as the healthy mode, which is also reflected in Figure 8.

Certain fault modes, such as SBAND_OverHeat, GPS_Lock_Failures, and RW_Tripped, have lower precision than other classes. The S-band XCVR has a limited operation period due to the short ground-contact window between the low-Earth-orbit satellite and the ground station. Therefore, the number of SBAND_OverHeat samples is relatively small, leading to lower precision. GPS_Lock_Failures can be detected when the PVT information within the OBC remains unchanged, but the algorithm requires a minimum number of corresponding telemetry samples within the windowed temporal segment $X_{t}$. Lastly, Figure 9 shows that RW_Tripped is often mistakenly identified as RW_NoResponse because the RW does not respond to the actuator controller when it is tripped or turned off.

Overall, these results show that the proposed model effectively captures both the spatial topology and temporal evolution patterns of satellite telemetry. Compared with traditional single-time-step CNN approaches, incorporating sliding-window temporal context significantly enhances inter-class separability and enables earlier identification of developing faults before they fully manifest.

The superior performance of the proposed framework can be attributed to three key design elements:

1. *Spatiotemporal representation learning*: By reshaping high-dimensional telemetry vectors into structured 2D grids, the model preserves latent correlations among heterogeneous sensors. The 3D convolutional architecture further exploits temporal continuity within sliding windows, enabling the extraction of evolution patterns that are essential for distinguishing subtle or gradually developing faults.
2. *Sliding-window modeling of fault progression*: Unlike point-wise classifiers that operate on individual time steps, the proposed window-based mechanism incorporates contextual temporal information. This allows the network to observe early fault signatures and progression trends before degradation becomes fully observable in the telemetry stream.
3. *Class-balanced learning for imbalanced distributions*: The integration of an effective-number-based weighting scheme mitigates the dominance of majority classes during training and improves the recall of rare fault types. This is particularly important in satellite health monitoring, where many failure modes occur infrequently but pose critical operational risks.

Collectively, these components enable the 3D-CNN architecture to learn robust, discriminative, and physically meaningful representations. This demonstrates its strong suitability for intelligent onboard health monitoring in small satellites operating under dynamic and resource-constrained conditions.

### 4.5. Ablation Study

To better understand the contribution of each key component, a unified ablation study was conducted from two perspectives:

1. ***Spatiotemporal modeling***: No-window, 2D-CNN, and 3D-CNN.
2. ***Imbalance-aware training***: Cross-Entropy (CE), CB weighting, and CB-Focal.

All variants followed the same data split, preprocessing settings, optimizer configuration, and training protocol. Only the component under study was changed.

Table 5 summarizes the results. Removing temporal context, i.e., No-window, leads to a clear performance drop, indicating that single-timestep telemetry snapshots are insufficient to characterize fault evolution. Replacing 3D convolution with 2D-CNN further degrades performance, with Accuracy dropping to 85.3%. This suggests that jointly learning temporal–spatial receptive fields is beneficial for capturing cross-parameter couplings that evolve over time. In addition, compared with standard cross-entropy, imbalance-aware objectives improve overall Accuracy, reflecting enhanced sensitivity to minority fault modes under severe class imbalance.

Table 5 shows that removing temporal context (No-window) leads to a clear performance drop. The results indicate that single-timestep telemetry snapshots are insufficient to characterize fault evolution. Replacing 3D convolution with a 2D-CNN further degrades performance. Accuracy drops to 85.3%, suggesting that jointly learning temporal–spatial receptive fields is beneficial for capturing cross-parameter couplings that evolve over time. In addition, compared with standard cross-entropy, imbalance-aware objectives improve the overall accuracy, reflecting enhanced sensitivity to minority fault modes under severe class imbalance.

### 4.6. Computational Efficiency

A quantitative efficiency comparison was conducted between the proposed model and representative baselines to evaluate computational complexity and practical feasibility. The number of parameters, FLOPs per telemetry window, model size, and inference latency per window were considered. All latency values were measured under the same workstation environment described in Section 4.1. The results are summarized in Table 6.

Table 6 shows that traditional and shallow models, such as SVM and MLP, have the lowest computational cost, with inference latencies of 0.042±0.005 ms/window and 0.057±0.003 ms/window, respectively. However, these methods mainly operate on flattened telemetry features and lack explicit spatial or spatiotemporal representation-learning capability. Therefore, although they are computationally efficient, their diagnostic performance is limited when handling high-dimensional satellite telemetry with temporal dependencies and cross-parameter coupling.

LSTM–RF and CNN–LSTM introduce temporal modeling while maintaining relatively low computational cost, requiring 38.40 MFLOPs and 42.15 MFLOPs per telemetry window, respectively. Their inference latencies are also lower than those of the proposed model. However, these methods generally perform temporal and spatial feature extraction in separate or sequential stages, resulting in lower Accuracy, Precision, and Recall, as shown in Table 3. In particular, CNN–LSTM first extracts spatial features from telemetry grids and then models temporal dependencies using recurrent units. In contrast, the proposed 3D-CNN performs joint temporal–spatial convolution within a unified feature-learning framework, allowing it to directly capture evolving cross-parameter interactions across consecutive telemetry grids.

The advanced spatiotemporal baselines, such as TCN–CNN, Transformer, and 3D-CNN-SE, provide stronger temporal or spatiotemporal modeling capability than conventional shallow models. TCN–CNN requires 86.40 MFLOPs and achieves an inference latency of 0.512±0.045 ms/window, while the Transformer model requires 125.30 MFLOPs with a latency of 0.724±0.081 ms/window. The attention-enhanced 3D-CNN-SE model has a computational cost of 224.15 MFLOPs and an inference latency of 0.915±0.102 ms/window. Compared with 3D-CNN-SE, the proposed 3D-CNN has slightly fewer parameters, a smaller model size, lower FLOPs, and shorter inference latency, while achieving better diagnostic performance as reported in Table 3. This indicates that the proposed residual 3D-CNN provides a more efficient spatiotemporal representation than simply adding an attention module to a 3D-CNN backbone.

Compared with 2D-CNN and ResNet, the proposed 3D-CNN has fewer trainable parameters and a smaller model size. The proposed 3D-CNN has 3,479,000 parameters and a model size of 13.278 MB, whereas 2D-CNN has 4,752,024 parameters and 18.141 MB, and ResNet has 21,288,558 parameters and 81.275 MB. However, the proposed 3D-CNN requires higher FLOPs and slightly longer inference latency than 2D-CNN because its convolutional kernels operate over both temporal and spatial dimensions. This additional computational cost is expected and is associated with the explicit modeling of local fault-evolution patterns, which enables the proposed method to achieve higher Accuracy, Precision, Recall, and F1-score than 2D-CNN, as shown in Table 3.

On the other hand, ResNet requires 3.67 GFLOPs per window and has an inference latency of 3.440±1.072 ms/window. The proposed 3D-CNN requires only 218.60 MFLOPs per window, with an inference latency of 0.886±0.094 ms/window. This indicates that the proposed model achieves a more favorable balance between diagnostic performance and computational efficiency than a deeper 2D residual network.

Overall, although the proposed 3D-CNN is not the lightest model among all compared methods, it provides a practical trade-off between computational cost and spatiotemporal diagnostic capability. Its average inference latency remains below 1 ms per telemetry window under the evaluated workstation environment, which is substantially shorter than the 60 s telemetry sampling interval used in this study. Therefore, the proposed model shows promising real-time processing potential for satellite health monitoring.

It should be emphasized that the reported computational profile was measured on a workstation rather than on flight-qualified onboard hardware. Therefore, these results should be interpreted as quantitative evidence of computational efficiency and preliminary real-time applicability, rather than as a complete demonstration of the feasibility of onboard deployment. Future work will further investigate embedded deployment, reduced-precision inference, pruning, model compression, and hardware-in-the-loop validation on representative onboard computing platforms. 

## 5. Conclusions

This paper proposes an imbalance-aware spatiotemporal fault diagnosis framework based on sliding-window segmentation and a lightweight residual three-dimensional convolutional neural network (3D-CNN) for system-level small-satellite telemetry. The proposed method converts high-dimensional telemetry vectors into subsystem- and function-informed 2D grids, stacks consecutive grids into voxel-like temporal windows, and applies 3D convolution to jointly capture local cross-parameter correlations and short-term temporal evolution patterns. In addition, an effective-number-based class-weighting strategy combined with Focal Loss is introduced to mitigate the severe imbalance between the dominant healthy state and rare fault classes.

Experimental results on the Lumelite telemetry dataset demonstrate that the proposed framework achieves superior Accuracy, Macro-Precision, Macro-Recall, and Macro-F1 score compared with traditional machine learning models, sequence-based models, 2D-CNN, and advanced spatiotemporal baselines. The class-wise recall results further show that the proposed imbalance-aware training strategy improves the recognition of several low-frequency fault types, indicating enhanced sensitivity to rare anomalies under a long-tailed distribution. Confusion matrix and t-SNE analyses also suggest that the learned spatiotemporal representations form more compact and separable feature clusters, supporting the effectiveness of the proposed temporal–spatial feature modeling strategy.

Although the proposed model shows promising inference latency, the evaluation was conducted on a workstation rather than on space-qualified onboard hardware. Therefore, the current results should be interpreted as preliminary computational evidence rather than a complete demonstration of onboard deployment feasibility.

Despite these advantages, the proposed framework still has several limitations. First, this study focuses on single-label fault identification, in which each telemetry segment is associated with a single dominant operational state. Second, the current validation is based on simulated Lumelite telemetry. Third, the current 2D telemetry embedding is manually defined according to subsystem and functional categories, rather than optimized using statistical correlations, causal dependencies, or physical topology.

Future work will evaluate the proposed method using hardware-in-the-loop tests with real telemetry and controlled fault injection on space-qualified system-on-module hardware. The present framework will also be further expanded for simultaneous multi-fault diagnosis. Furthermore, new processing methods, such as correlation-aware, topology-aware, and graph-based telemetry representations, will be explored to improve physical interpretability and cross-parameter dependency modeling.

## Figures and Tables

**Table 1:** Sensitivity analysis of the sliding-window length *L* and CB-Focal loss parameters β and γ.

No.	*L*	β	γ	Accuracy (%)	Precision (%)	Recall (%)	F1-Score (%)
1	3	0.990	1.0	91.24	93.10	85.12	88.93
2	3	0.999	2.0	92.85	94.20	87.80	90.89
3	5	0.990	2.0	93.40	95.80	87.50	91.46
4	5	0.999	1.0	93.95	96.10	88.40	92.09
5	5	0.999	2.0	94.62	96.45	90.57	93.00
6	5	0.999	3.0	94.10	94.80	91.20	92.97
7	5	0.9999	2.0	93.80	94.15	92.10	93.11
8	7	0.999	2.0	94.25	96.12	89.90	92.91
9	7	0.9999	3.0	93.55	93.80	91.45	92.61

**Table 2:** Hyperparameter settings used for training the proposed model.

Category	Hyperparameter	Value/Setting
Training Configuration	Optimizer	AdamW
	Initial Learning Rate	$1\times 10^{-3}$
	Weight Decay	$3\times 10^{-4}$
	Batch Size	128
	Epochs	100
	Learning Rate Scheduler	Cosine Annealing
	Early Stopping Patience	20
Model Architecture	Window Length *L*	5
	Window Stride	1
	Grid Size (H,W)	40×40
	3D-CNN Dropout Rates	0.30/0.30/0.20
Imbalance Handling	Focal Loss γ	2.0
	Effective Number β	0.999

**Table 3:** Performance comparison between the proposed model and baseline fault diagnosis models on the Lumelite satellite telemetry dataset.

Method	Accuracy (%)	Precision (%)	Recall (%)	F1-Score (%)
Proposed	**94.62**	**96.45**	**90.57**	**93.00**
LSTM–RF [28]	71.82	74.10	62.34	67.65
SVM [29]	75.46	77.98	65.71	71.22
2D-CNN [30]	78.23	80.51	73.84	77.00
CNN-LSTM [31]	79.35	81.27	75.92	78.52
ResNet [32]	82.14	84.09	77.38	80.61
3D-CNN-SE [33]	89.85	91.30	85.22	88.15
TCN-CNN [34]	85.22	87.35	80.18	83.61
Transformer [35]	87.40	89.15	82.60	85.75

**Table 4:** Class-wise Recall (%) of different methods on the test set.

	Method	Proposed	LSTM-RF	SVM	2D-CNN	CNN-LSTM	ResNet
Class							
0		98.3	97.9	98.0	97.0	96.5	96.0
1		88.5	3.1	12.5	65.4	82.1	91.2
2		98.0	92.0	91.5	90.5	90.0	89.5
3		99.5	74.6	80.2	88.2	94.5	96.6
4		97.4	0.0	0.0	0.0	0.0	0.0
5		96.8	69.6	75.4	88.6	95.2	98.4
6		63.4	84.3	83.8	82.5	81.8	81.1
7		100.0	97.2	98.1	98.8	99.2	99.4
8		100.0	98.3	98.8	99.5	99.8	100.0
9		99.9	93.4	94.5	96.4	97.8	98.5
10		55.5	0.0	0.0	0.0	0.0	0.0
11		100.0	97.9	98.2	98.8	99.2	99.9
12		100.0	99.5	99.6	99.8	99.9	100.0
13		99.6	14.6	35.2	72.4	88.6	93.9
14		95.0	89.6	90.2	91.8	92.5	93.2
15		97.6	88.9	89.5	91.5	93.2	94.2
16		86.1	2.2	15.4	45.2	55.4	62.0
17		83.1	39.5	80.2	41.8	43.0	44.0
18		93.7	24.1	45.6	78.4	91.2	96.7
19		100.0	95.8	96.5	97.5	98.2	98.5
20		97.1	40.6	43.5	49.2	52.6	55.4
21		84.4	0.0	0.0	0.0	0.0	0.0
22		78.2	13.0	12.5	45.6	56.8	65.0
23		60.9	0.0	0.0	5.2	12.6	21.7

**Table 5:** Unified ablation study of the proposed framework with comprehensive metrics.

Variant	Accuracy (%)	Precision (%)	Recall (%)	F1-Score (%)
No-window + CB-Focal	72.41	70.15	68.30	69.21
2D-CNN + CB-Focal	84.32	83.50	82.11	82.80
3D-CNN + CE	88.60	89.12	85.45	87.25
3D-CNN + CB	90.10	89.85	88.20	89.02
**3D-CNN + CB-Focal**	**94.62**	**96.45**	**90.57**	**93.00**
**(Proposed)**				

**Table 6:** Computational complexity comparison between the proposed model and baseline models.

Method	Parameters	FLOPs	Model Size (MB)	Latency (ms)
SVM	–	0.05 M	0.240	0.042±0.005
MLP	203,438	0.20 M	0.776	0.057±0.003
LSTM–RF	854,200	38.40 M	3.260	0.412±0.062
CNN–LSTM	902,616	42.15 M	3.447	0.366±0.053
TCN–CNN	1,245,600	86.40 M	4.752	0.512±0.045
Transformer	2,150,000	125.30 M	8.204	0.724±0.081
3D-CNN-SE	3,586,400	224.15 M	13.682	0.915±0.102
2D-CNN	4,752,024	145.24 M	18.141	0.664±0.177
ResNet	21,288,558	3.67 G	81.275	3.440±1.072
Proposed	3,479,000	218.60 M	13.278	0.886±0.094

## Data Availability

The simulated satellite system training and testing datasets that support the conclusions of this article will be made available by the authors upon request.

## Abbreviations

The following abbreviations are used in this manuscript:

3D	Three-Dimensional
ADCS	Attitude Determination and Control Subsystem
ANT	Antenna
AOM	Array Optimizer Module
BATT	Battery
BMM	Battery Management Module
CIM	Communication Interface Module
CNN	Convolutional Neural Network
COMM	Communication Subsystem
DSP	Digital Signal Processor
EPS	Electronic Power Subsystem
GELU	Gaussian Error Linear Unit
GPS	Global Positioning System
IMU	Inertial Measurement Unit
LSTM	Long Short-Term Memory
MT	Magnetic Torquer
OBC	Onboard Computer Subsystem
PCDM	Power Control and Distribution Module
PV	Photovoltaic
ResNet	Residual Neural Network
RW	Reaction Wheel
SVM	State Vector Machine
t-SNE	t-distributed Stochastic Neighbor Embedding
UHF	Ultra-High Frequency
XCVR	Transceiver

## References

[1] Sweeting M.N. (2018). Modern Small Satellites-Changing the Economics of Space. Proc. IEEE.

[2] Bouwmeester J., Menicucci A., Gill E. (2022). Improving CubeSat reliability: Subsystem redundancy or improved testing?. Reliab. Eng. Syst. Saf..

[3] Suo M., Xing J., Ragulskis M., Dong Y., Zhang Y., Lu C. (2024). Fault diagnosis of satellite power system based on unsupervised knowledge acquisition and decision-making. Adv. Eng. Inform..

[4] Xia H., Meng T. (2025). A versatile feature selection learning method for satellite attitude control system fault diagnosis with limited data. Neurocomputing.

[5] Zhao J., Perrin O., Ahangarpour A., Pan J. Measuring the OneWeb Satellite Network. Proceedings of the 2025 9th Network Traffic Measurement and Analysis Conference (TMA).

[6] Foo K.J.E., Tissera M.S.C., Low K.S., Srivastava A. (2025). FLITESIM: A Comprehensive Verification and Validation Environment for Small Satellite Attitude Determination and Control Systems. IEEE Access.

[7] Venkatasubramanian V., Rengaswamy R., Yin K., Kavuri S.N. (2003). A review of process fault detection and diagnosis: Part I: Quantitative model-based methods. Comput. Chem. Eng..

[8] Venkatasubramanian V., Rengaswamy R., Kavuri S.N., Yin K. (2003). A review of process fault detection and diagnosis: Part II: Qualitative models and search strategies. Comput. Chem. Eng..

[9] Venkatasubramanian V., Rengaswamy R., Kavuri S.N., Yin K. (2003). A review of process fault detection and diagnosis: Part III: Process history based methods. Comput. Chem. Eng..

[10] Shangguan D., Chen L., Ding J. (2020). A Digital Twin-Based Approach for the Fault Diagnosis and Health Monitoring of a Complex Satellite System. Symmetry.

[11] Zhang Z.H., Li S., Yan H., Fan Q.Y. (2019). Sliding mode switching observer-based actuator fault detection and isolation for a class of uncertain systems. Nonlinear Anal. Hybrid Syst..

[12] Baldi P., Blanke M., Castaldi P., Mimmo N., Simani S. (2019). Fault diagnosis for satellite sensors and actuators using nonlinear geometric approach and adaptive observers. Int. J. Robust Nonlinear Control.

[13] Liu G., Zhang W., Xu H., Wu Y. (2019). A Rule-Base-Driven Fault Diagnosis System for the Ground Segment of Satellite Communication Systems. IEEE Access.

[14] Long M., Zhu H., Zhang G., He W. (2024). Aerospace Equipment Fault Diagnosis Method Based on Fuzzy Fault Tree Analysis and Interpretable Interval Belief Rule Base. Mathematics.

[15] Lee T., Choi Y., Kim Y. (2021). Hybrid Fault Detection and Severity Estimation for Complex Spacecraft Propulsion Systems Using XGBoost and Rule-Based Reasoning. Aerosp. Sci. Technol..

[16] Chen Z. Universal CubeSat Platform Design Technique. Proceedings of the 2nd International Conference on Mechanical, Material and Aerospace Engineering.

[17] Gallon R., Schiemenz F., Menicucci A., Gill E. (2024). Convolutional Neural Network Design and Evaluation for Real-Time Multivariate Time Series Fault Detection in Spacecraft Attitude Sensors. Adv. Space Res..

[18] Chen J., Pi D., Wu Z., Zhao X., Pan Y., Zhang Q. (2021). Imbalanced Satellite Telemetry Data Anomaly Detection Model Based on Bayesian LSTM. Acta Astronaut..

[19] Xiang G., Miao J., Cui L., Hu X. (2022). Intelligent Fault Diagnosis for Inertial Measurement Unit through Deep Residual Convolutional Neural Network and Short-Time Fourier Transform. Machines.

[20] Xu Y., He D., Sun H., Jin Z., Zhao M. (2026). Self-supervised learning for train bearing fault diagnosis based on time–frequency dual domain prediction. Struct. Health Monit..

[21] He D., Xu Y., Sun H., Jin Z., Zhao M. (2025). Self-supervised learning for vehicle bearing fault diagnosis based on time–frequency dual-domain contrast and fusion. Nonlinear Dyn..

[22] Tang H., Li J., Feng W., Chen P., Xue H. (2025). Towards machinery incremental fault diagnosis based on inverted transformer lifelong learning with learnable pruning mechanism. Eng. Appl. Artif. Intell..

[23] Tang H., Zu X., Guo Y., Jiang X., Wang J., Lin R., Xue H., Wang H. (2026). A novel incremental method with dynamic learnable pruning mechanism for low-speed machinery fault diagnosis. Eng. Appl. Artif. Intell..

[24] Huang R., Li J., Liao Y., Chen J., Wang Z., Li W. (2021). Deep Adversarial Capsule Network for Compound Fault Diagnosis of Machinery Toward Multidomain Generalization Task. IEEE Trans. Instrum. Meas..

[25] Li W., Lan H., Chen J., Feng K., Huang R. (2023). WavCapsNet: An Interpretable Intelligent Compound Fault Diagnosis Method by Backward Tracking. IEEE Trans. Instrum. Meas..

[26] Zhang S., Zhou J., Ma X., Pirttikangas S., Yang C. (2024). TSViT: A Time Series Vision Transformer for Fault Diagnosis of Rotating Machinery. Appl. Sci..

[27] Xu Y., Li S., Li Z., Wu K., Huang R., Sun B., Ji J.C. (2026). Time–frequency fully-connected graph neural network: An effective multiscale spatiotemporal dependency learning method for multisource machine fault diagnosis. Adv. Eng. Inform..

[28] Wang B., Zhou Y., Shi Y., Goh S.T., Rai A. Multi-Label Fault Detection in Small Satellite ADCS Using Hybrid Random Forest and LSTM Algorithm. Proceedings of the AIAA Scitech.

[29] Zhao S., Zhang Y.C. (2008). SVM Classifier Based Fault Diagnosis of the Satellite Attitude Control System. 2008 International Conference on Intelligent Computation Technology and Automation (ICICTA).

[30] Zhao H., Liu M., Sun Y., Chen Z., Duan G., Cao X. (2024). Automated Design of Fault Diagnosis CNN Network for Satellite Attitude Control Systems. IEEE Trans. Cybern..

[31] Hanyu L., Chengrui L., Wenjing L., Wenbo L., Heyu X. (2024). Intelligent fault diagnosis method of spacecraft control system based on sequence data-image mapping. Math. Found. Comput..

[32] He K., Zhang X., Ren S., Sun J. Deep Residual Learning for Image Recognition. Proceedings of the IEEE Conference on Computer Vision and Pattern Recognition (CVPR).

[33] Wang T., Yin L. (2024). A hybrid 3DSE-CNN-2DLSTM model for compound fault diagnosis of wind turbines with SCADA data. Expert Syst. Appl..

[34] Hong L., Li D., Gao L. (2022). A novel CNN-TCN-TAM classification model based method for fault diagnosis of chiller sensors. Proceedings of the 41st Chinese Control Conference (CCC).

[35] Jin Y., Hou L., Chen Y. (2022). A Time Series Transformer based method for the rotating machinery fault diagnosis. Neurocomputing.

[36] Rai A., Tissera M.S.C., Srivastava A., Goh S.T., Sii Z.Y., Foo K.J.E., Yuan F., Tariq M.A., Low K.S. Lumelite-4: A Satellite Built with Modularity and Scalability Concept. Proceedings of the 35th International Symposium on Space Technology and Science.

[37] Cheong J.W., Southwell B.J., Andrew W., Aboutanios E., Lam C., Croston T., Li L., Green S., Kroh A., Glennon E.P. (2020). A robust framework for low-cost Cubesat scientific missions: In-orbit recovery, results and lessons learned from UNSW-EC0. Space Sci. Rev..

[38] Motahhir S., Hammoumi A.E., Ghzizal A.E. (2019). The most used MPPT algorithms: Review and the suitable low-cost embedded board for each algorithm. J. Clean. Prod..

[39] Tissera M.S.C., Foo K.J.E., Low K.S., Goh S.T., Tan R.D. (2023). ROEKF-MPC Estimator for Satellite Attitude and Gyroscope Bias Estimation. IEEE Trans. Aerosp. Electron. Syst..

[40] Goh S.T., Tissera M.S.C., Tan R.D., Srivastava A., Low K.S., Lim L.S. (2022). Simplex Back Propagation Estimation Method for Out-of-Sequence Attitude Sensor Measurements. Sensors.

[41] Vallado D.A., Cefola P.J. TWO-LINE ELEMENT SETS – PRACTICE AND USE. Proceedings of the 63rd International Astronautical Congress.

[42] Shah V., Youngblood N. (2025). Leveraging Continuously Differentiable Activation for Learning in Analog and Quantized Noisy Environments. IEEE J. Sel. Top. Quantum Electron..

[43] Hendrycks D., Gimpel K. (2016). Gaussian error linear units (gelus). arXiv.

[44] Bishop C.M. (2006). Pattern Recognition and Machine Learning.

[45] Cui Y., Jia M., Lin T.Y., Song Y., Belongie S. Class-Balanced Loss Based on Effective Number of Samples. Proceedings of the IEEE/CVF Conference on Computer Vision and Pattern Recognition (CVPR).

[46] Lin T.Y., Goyal P., Girshick R., He K., Dollár P. Focal Loss for Dense Object Detection. Proceedings of the IEEE International Conference on Computer Vision (ICCV).

[47] Cao K., Wei C., Gaidon A., Arechiga N., Ma T. Learning Imbalanced Datasets with Label-Distribution-Aware Margin Loss. Proceedings of the Advances in Neural Information Processing Systems (NeurIPS).

[48] Ren J., Yu C., Sheng S., Ma X., Zhao H., Yi S., Li H. Balanced Meta-Softmax for Long-Tailed Visual Recognition. Proceedings of the Advances in Neural Information Processing Systems (NeurIPS).

[49] Grandini M., Bagli E., Visani G. (2020). Metrics for Multi-class Classification: An Overview. arXiv.

[50] van der Maaten L., Hinton G. (2008). Visualizing Data using t-SNE. J. Mach. Learn. Res..

